# Novel Lipid-Based Formulation to Enhance Coenzyme Q10 Bioavailability: Preclinical Assessment and Phase 1 Pharmacokinetic Trial

**DOI:** 10.3390/pharmaceutics17040414

**Published:** 2025-03-25

**Authors:** Andrea Fratter, Alessandro Colletti, Giancarlo Cravotto, Marzia Pellizzato, Adele Papetti, Vanessa Pellicorio, Chiara Bolego, Marco Simiele, Antonio D’Avolio, Andrea Cignarella

**Affiliations:** 1 Department of Pharmaceutical and Pharmacological Sciences (DSFarm), University of Padova, 35129 Padua, Italy; 2 Italian Society of Nutraceutical Formulators (SIFNut), 31100 Treviso, Italyandrea.cignarella@unipd.it (A.C.); 3 Department of Drug Sciences, University of Torino, 10124 Turin, Italy; 4 Department of Drug Sciences, University of Pavia, 27100 Pavia, Italy; 5 CoQua Lab, 10149 Turin, Italy; 6 Department of Medical Sciences, University of Turin, 10124 Turin, Italy; 7 Department of Medical and Surgical Sciences, University of Padova, 35122 Padua, Italy

**Keywords:** coenzyme Q10, lipid-based drug delivery system, Caco-2, bioaccessibility, bioavailability

## Abstract

**Background:** Nutraceuticals represent a strategy for maintaining health and constitute a brilliant market in Italy and across Europe. However, the absence of strict regulations regarding formulation requirements highlights a critical issue: their poor bioavailability. An example is coenzyme Q10 (CoQ10), a quinone known for its potential as a mitochondrial protective agent but characterized by low intestinal absorption. CoQ10 is a hydrophobic molecule with high molecular weight and poor water solubility, factors that significantly limit its intestinal bioaccessibility and, consequently, its oral bioavailability. **Objectives**: In this context, the present study describes a novel formulation designed to enhance CoQ10 bioaccessibility through in situ emulsification upon contact with gastroenteric fluids. This technology, termed Lipid-Based Auto-Emulsifying Drug Delivery System (LiBADDS), is unique because it combines a medium-chain triglyceride (MCT), a long-chain fatty acid, conjugated linoleic acid (CLA) with a high HLB solubilizer, Polysorbate 80 (PS80), and a sodium octenyl succinate starch derivative (SOS), which can create a nanometric emulsion simply by aqueous dispersion and upon contact with gastrointestinal fluids. This phenomenon promotes the prompt dispersion of CoQ10 and its rapid translocation into the serosal compartment of the intestinal epithelium. **Methods:** Its efficacy was evaluated in vitro through the Caco-2 cellular model and in vivo through a crossover study on healthy volunteers, measuring pharmacokinetic parameters such as AUC, C_max_, T_max_, ΔAUC, and ΔC_max_. **Results:** Overall, LiBADDS demonstrated a significant improvement in both the bioaccessibility and bioavailability of CoQ10 compared to the unformulated substance. **Conclusions:** LiBADDS showed to be a promising tool to improve CoQ10 bioavailability by enhancing its bioaccessibility.

## 1. Introduction

Coenzyme Q10 (CoQ10) is an isoprenoid quinone present in the mitochondrial respiratory chain, where it acts as an electron carrier essential for ATP synthesis. Its concentration is particularly high in tissues with significant mechanical activity, such as skeletal muscles and the myocardium [1,2,3].

In recent years, CoQ10 has garnered attention from clinicians and pharmacologists due to its radical-scavenging properties, particularly in the context of antineoplastic therapies involving anthracyclines [4,5,6,7,8,9]. These drugs are associated with severe adverse effects, including cardiomyopathy resulting from mitochondrial dysfunction in cardiomyocytes [10,11,12]. Additionally, CoQ10 has been investigated for its potential role in preventing statin-induced myopathy, although clinical evidence remains inconclusive [13,14,15,16,17,18].

CoQ10 is widely used in high-turnover products classified as dietary supplements, given its safety profile even at high doses and over extended periods. Its deficiency is particularly prevalent among elderly individuals and patients undergoing long-term statin therapy. However, its classification as a dietary supplement poses challenges, particularly concerning its poor intestinal absorption due to its hydrophobicity and high molecular weight, which limit its solubility in aqueous enteric fluids [19,20,21,22]. As extensively documented in the literature, CoQ10 exhibits low bioavailability primarily due to its poor intestinal bioaccessibility. Consequently, only formulations that ensure rapid and complete aqueous dispersion can achieve acceptable absorption rates [23,24].

The development of advanced formulations aimed at improving CoQ10’s enteric bioaccessibility is crucial for maximizing its therapeutic efficacy, particularly in mitigating statin-induced myopathy and, potentially, in reducing or preventing anthracycline-induced cardiotoxicity. Various formulation strategies have been explored to enhance CoQ10 absorption, including encapsulation in cyclodextrins, nano-dispersions, nano-capsules, Self-Emulsifying Drug Delivery Systems (SEDDS), and Self-Nano-Emulsifying Drug Delivery Systems (SNEDDS) [25,26,27,28,29,30]. More recently, the development of lipid-based delivery systems that facilitate the lymphatic transport of lipophilic molecules has gained significant traction among researchers.

Among these, Lipid-Based Auto-Emulsifying Drug Delivery Systems (LiBADDSs) appear particularly promising for CoQ10 delivery, as its physiological absorption occurs through incorporation into lipoproteins, particularly chylomicrons [31]. This study presents a novel LiBADDS formulation in which CoQ10 is dissolved in a mixture containing medium-chain triglycerides (MCTs), the polyoxyethylene sorbitan ester Polysorbate 80 (PS80), and the monounsaturated fatty acid conjugated linoleic acid (CLA). Literature reports suggest that CLA can facilitate the incorporation of hydrophobic compounds into lipoproteins, thereby improving their absorption [32].

To ensure effective emulsification upon exposure to intestinal fluids, the oil-based solution was adsorbed onto a solid surfactant agent derived from starch (sodium octenyl succinate starch). The LiBADDS system underwent simulated gastrointestinal digestion, followed by the characterization of intestinal dispersion via Dynamic Light Scattering (DLS). The bioaccessible fraction (BF) was subsequently evaluated using an in vitro human intestinal Caco-2 cell model to assess permeation. Finally, a pharmacokinetic study was conducted in healthy volunteers using a crossover design to determine whether the LiBADDS formulation improved CoQ10 bioavailability compared to the unformulated compound.

Overall, the CoQ10 LiBADDS system demonstrated better bioaccessibility and bioavailability, indicating that the formation of an in situ nanoemulsion or, more likely, a spontaneous microemulsion, promoted by the LiBADDS technology facilitated the dispersion of CoQ10 and simultaneously favored the incorporation of CoQ10 into lipoproteins and chylomicrons, as shown by the high internalization rate in Caco-2 cells and the undetectable concentration of CoQ10 in the basolateral compartment. This technology offers an innovative and novel delivery method compared to those previously described, particularly due to the simultaneous presence of several emulsifiers. PS80, a well-known solubilizing and emulsifying agent, a long-chain fatty acid (CLA) acting as an ionic emulsifier in the intestinal environment after salt formation, and an octenylsuccinate derivative of starch. This combination of emulsifiers is further enhanced in its surfactant capacity in the small intestine by the action of bile salts and the mono- and diglycerides of fatty acids, which create a mixed emulsion with oil droplets possessing high elasticity and deformability.

From the data collected, CoQ10 LiBADDS showed enhanced bioavailability in comparison to unformulated CoQ10 in particular in the interval 2–6 h in which the increase in AUC reached 40%. Also noteworthy are the data related to the ΔAUC_2–6h_, which stood at 200% in favor of LiBADDS.

## 2. Materials and Methods

### 2.1. Materials

Veremul T 80 (Polysorbate 80) was obtained from Eigenmann-Veronelli (Milan, Italy), while Tocopherol and Tonalin A 80 (conjugated linoleic acid carboxylic) were supplied by BASF (Cesano Maderno, MB, Italy). Coenzyme Q10 was purchased from Beck Ingredients (Barcelona, Spain). Cleargum CO 001 (sodium starch octenyl succinate) was acquired from Roquette (Alessandria, Italy), and Glucidex D19 from Faravelli SPA (Nerviano, MI, Italy). Amorphous silica was obtained from Brenntag (Milan, Italy). Fasted State Simulated Intestinal Fluids (FaSSIF) and Fasted State Simulated Gastric Fluids (FSSGF) were supplied by Biorelevant (London, UK). Pepsin and pancreatin 4 × USP (from porcine gastric mucosa), methanol, and ethyl acetate were sourced from Merck (Rome, Italy).

### 2.2. RP-HPLC-DAD Analysis

An HPLC analysis was performed using an Agilent 1200 system (Waldbronn, Germany), equipped with an online degasser, quaternary pump, autosampler, column heater, and diode array detector (DAD). The separation was carried out on a Gemini^®^ C18 analytical column (150 × 2.0 mm i.d., 5 µm, Phenomenex, Torrance, CA, USA). The chromatographic conditions were as follows:

- Column temperature: 25 °C;
- Flow rate: 0.3 mL/min;
- Injection volume: 20 µL;
- Detection wavelength: 275 nm;
- Mobile phase: 55% acetonitrile (ACN), 40% tetrahydrofuran (THF), 5% water;
- Elution mode: isocratic;
- Run time: 12 min;
- Limit of detection (LOD): 0.05 µg/mL;
- Limit of quantification (LOQ): 0.25 µg/mL.

A standard calibration curve was generated using CoQ10 diluted in the HPLC mobile phase, with final concentrations ranging from 0.5 to 150 µg/mL. The calibration curve is provided in the Supplementary Materials (Figure S1).

### 2.3. Preparation of CoQ10-Loaded LiBADDS Powder

A total of 2 g of Coenzyme Q10 was dispersed in a pre-warmed liquid phase at 50 °C, consisting of 4 g of Labrafac Lipophile WL 1349, 12 g of Veremul T80, 2 g of Tonalin A 80, and 0.25 g of Tocopherol. The mixture was continuously stirred at 100 rpm using a magnetic stirrer in the dark until complete solubilization of CoQ10, resulting in a clear orange phase. The CoQ10 solution was then poured onto a solid mixture composed of 392.75 g of Glucidex D19 (maltodextrins) and 50 g of Cleargum CO 001 (sodium starch octenyl succinate) under mechanical stirring to ensure uniform absorption of the liquid phase. The resulting wet powder was subsequently blended with 35 g of amorphous silica to obtain a free-flowing dry powder, with a total mass of 500 g (Table 1).

### 2.4. Simulated Digestion of CoQ10-Loaded LiBADDS Sachets

One sachet was dispersed in 50 mL of FSSGF (pH 1.6) and incubated at 37 °C under continuous stirring (100 rpm) for 2 h in the presence of pepsin (2000 U/mL). Following gastric incubation, 50 mL of FaSSIF was added, and the pH was adjusted to 6.5 using a 10% sodium bicarbonate solution (*w*/*v*), following the two-stage digestion protocol described by Geboers et al. [33]. Pancreatin 4X USP was then introduced at a final concentration of 10 mg/mL, and the mixture was stirred for an additional hour at 37 °C. The final FSSGF/FaSSIF dispersion was centrifuged at 10,000 rpm at 37 °C for 15 min. The supernatant (bioaccessible fraction) was analyzed for particle size distribution using Dynamic Light Scattering (DLS), while an aliquot was set aside for enteric absorption testing using the Caco-2 in vitro model. The insoluble pellet was collected for CoQ10 quantification via RP-HPLC-DAD.

### 2.5. Particle Size Determination of CoQ10 Dispersed Phase Using DLS

The Dynamic Light Scattering (DLS) analysis was performed using a Zetasizer Nano-ZS ZEN 3600 (Malvern, UK). The particle size range was set between 0.6 nm and 6.0 µm. Measurements were conducted at 25 °C using disposable polystyrene cuvettes (10 × 10 × 45 mm), with water as the dispersing medium (refractive index: 1.330, viscosity: 0.8872 cp). Each sample required 1 mL for analysis.

### 2.6. Calculation of CoQ10 Bioaccessible Fraction

The supernatant obtained from Section 2.4 was mixed with an equal volume of ethyl acetate to break down micelles and extract CoQ10 in both reduced and oxidized forms. The organic phase was evaporated using a rotary evaporator, and the residue was dissolved in 4 mL of a mixture containing 2 mL of ferric chloride (0.01% ethanolic solution) and 2 mL of the RP-HPLC mobile phase to induce complete oxidation of ubiquinol to ubiquinone. The resulting sample was analyzed via RP-HPLC-DAD. The insoluble pellet was dissolved in 1 mL of ferric chloride (0.01% ethanolic solution) and 1 mL of the HPLC mobile phase, vortexed for 2 min, and centrifuged at 5000 rpm for 10 min at room temperature. The extraction process was repeated twice until a colorless pellet was obtained [34]. The combined supernatants were analyzed by RP-HPLC-DAD. The bioaccessible fraction (BF) was calculated using Equation (1):

BF = (CoQ10_mic_/CoQ10_tot_) × 100 (1)

where BF = bioaccessible fraction, CoQ10_mic_ = fraction of CoQ10 encapsulated in the supernatant, CoQ10_tot_ = total CoQ10 content recovered by RP-HPLC-DAD analysis.

### 2.7. Cell Culture Materials

The high-glucose Dulbecco’s Modified Eagle Medium (DMEM), Roswell Park Memorial Institute (RPMI) 1640 Medium, Hanks’ Balanced Salt Solution (HBSS), non-essential amino acids (NEAA), L-glutamine, penicillin–streptomycin mix, and Lucifer Yellow were purchased from Sigma-Aldrich (St. Louis, MO, USA). Caco-2 human colorectal adenocarcinoma cells (ATCC^®^ HTB-37™) were obtained from ATCC (Manassas, VA, USA). The CellTiter 96^®^ Aqueous One Solution Cell Proliferation Assay (MTS) was sourced from Promega (Madison, WI, USA). Transwell^®^ inserts were purchased from Millipore (Burlington, MA, USA) and fetal bovine serum (FBS) from Euroclone (Milan, Italy).

### 2.8. In Vitro Model of Intestine

The intestinal absorption of CoQ10 contained in CoQ10 LiBADDS 027 formulations was assessed using a human model of enteric epithelium in vitro, based on human cells of adenocarcinomas Caco-2 (ATCC, HTB-37™) organized in functional monolayer placed in Transwell inserts. The Transwell inserts are characterized by two compartments separated by a microporous membrane: the apical or luminal and basolateral or serosal. The monolayers composed of Caco-2 cells growing on the surface of microporous membranes consist of polarized cells with morpho-functional features typical of human enterocytes like the presence of microvilli, tight junctions, and efflux ATP-binding cassette (ABC) such as P-glycoprotein (P-gp). Briefly, Caco-2 cells were seeded at a density of 1.5 × 10^5^ cells/cm^2^ on 1 μm pore size Transwell^®^ polytetrafluoroethylene inserts and left to mature and differentiate for 21 days. Absorption experiments were performed at day 21 post-seeding.

### 2.9. Caco-2 Cell Culture

The Caco-2 cells were seeded in an adhesion flask in a 120-cell complete medium (high-glucose DMEM, 10% heat inactivated FBS, 1% non-essential amino acids, 4 mM L-glutamine, and 1% penicillin/streptomycin mix) at a density of 2 × 10^3^ cell/cm^2^ and kept at 37 °C and 5% CO_2_ in a humidified incubator. Cells were sub-cultivated by trypsinization every 7 d when 80–90% confluent and seeded at a density of 2000 cells/cm^2^. The medium was refreshed every other day.

### 2.10. Evaluation of the Formulations Impact on Enteric Cell Viability

The impact of digested formulations on the viability of enteric epithelium was assessed through a dose–response curve. After the digestive process, the supernatant (bioaccessible fraction), derived from digestion of the formulations, was diluted in digestive fluids. Different concentrations of supernatants were added to the apical compartment of the Caco-2 mono-layer, while the basolateral compartment received Hank’s Balanced Salt Solution Buffer (HBSS) enriched with 1% Bovine Serum Albumin (BSA) or Polyphosphate Saline Buffer (PBS). As a negative control, digestive fluids without RES and CoQ10 were used. After 3 h of incubation, the vitality of enteric epithelium was assessed through a 3-(4,5-dimethylthiazol-2-yl)-5-(3-carboxymethoxyphenyl)-2-(4-sulfophenyl)-2H-tetrazolium (MTS) assay, according to the manufacturer’s instructions. The MTS assay is based on the reduction of MTS tetrazolium compound from the vital cells in the presence of phenazine methosulfate to generate the colored compound formazan that can be titrated by measuring absorbance at 490 nm with a microplate reader (Synergy4, Biotek, Agilent Stevens Creek Blvd. Santa Clara, CA, USA). Cell viability (%) was expressed as the ratio of absorbance in the treated groups to that in the control (untreated) group. Meanwhile, the impact of the bioaccessible fractions of analyzed formulations on the monolayer integrity was assessed by measuring trans-epithelial electrical resistance (TEER) and Lucifer Yellow (LY) Papp and flux. A concentration of 30 mM was nontoxic and was chosen to assess permeability experiments.

### 2.11. Integrity of Barrier Function in the Model of Enteric Epithelium

The barrier integrity was assessed by measuring the TEER of the monolayer with an ERS2 Voltmeter 273 (Millipore) equipped with a chopstick electrode and by evaluating its permeability LY, a polar tracer unable to permeate across intact tight junctions. Permeability was measured by adding 0.2 mL of a 100 μg/mL solution of LY in HBSS in the apical compartment and 0.6 mL of HBSS in the basolateral compartment. After 1 h of incubation, the basolateral fractions were collected and the fluorescence intensity at 490 nm was determined with a microplate reader (Synergy4, Biotek, Agilent Stevens Creek Blvd. Santa Clara, CA, USA). To convert the rate of fluorescence in LY concentration (μg/mL), a calibration curve was obtained in the range from 0 to 12.5 μg/mL according to Table 2 (fluorescence values between 100 and 12.5 μg/mL were excluded since the fluorescence signal at these concentrations tends to be saturated).

### 2.12. Evaluation of CoQ10 Permeation Through the Enteric Epithelium

At the end of the digestive simulation process, the highest nontoxic concentration of bioaccessible fraction (supernatant) was added to the apical compartment of in vitro enteric epithelium. CoQ10-free digestive fluids were used as a control, and HBSS added with 1% BSA or PBS was applied to the basolateral compartment. After 3 h of exposure, the apical and basolateral fractions were recovered and their CoQ10 content assessed by an RP-HPLC analysis. The same procedure was adopted to assess permeation from basolateral to apical side. The apparent permeability coefficient (*P_app_*, cm/min) was calculated, in case of nonlinear model fitting, according to the following non-sink Artursson’s equation [35,36]:

CR(t) = [M/(V_D_ + V_R_)] + (CR_0_ − [M/(V_D_ + V_R_)])e(−*P_app_* A (1/V_D_ + 1/V_R_) t) (non-linear curve fitting equation) (2)

where V_D_ is the volume of the donor compartment, VR is the volume of the receiver compartment (cm^3^), A is the area of the membrane (cm^2^), M is the total amount of substance in the system, CR(0) is the concentration of the substance in the receiver compartment at the start of the time interval, and CR(t) is the substance concentration at time t measured from the start of the time interval.

The apparent permeability coefficient (*P_app_*, cm/min) was calculated, in case of steady state flux, according to Equation (3):

*P_app_* = (dQ/dt) × 1/(A × C_0_) (3)

where dQ/dt is the steady-state flux of the molecule transported through the monolayer during the incubation (mM/min), A is the area of the membrane (cm^2^), and C_0_ is the initial concentration of LY in the apical compartment.

### 2.13. Cellular Uptake

At the end of the permeation experiment, the cells placed on Transwell inserts, were washed three times with HBSS buffer or PBS, then a 0.25% trypsin solution was added, and they were incubated at 37 °C for 10 min. After that, cells and semipermeable membrane were carefully detached from the filter with a scalpel, collected into Eppendorf tube and 1 mL of PBS was added with 1% Triton ×100. After sonication in a water-bath sonicator for 10 min, the mixture was centrifugated at 10,000× *g* for 5 min, and 60 μL of the supernatant was dried under a nitrogen flux. The residue was dissolved with 30 μL of ferric chloride (0.01% ethanolic solution) and 30 μL of the HPLC mobile phase, sonicated, and centrifuged at 14,000 rpm for 10 min. The supernatant was finally analyzed by HPLC. Cell protein content was determined by a BCA assay.

### 2.14. Bioavailability Assessment

To verify whether the increase in bioaccessibility of CoQ10 induced by the LiBADDS technology, as confirmed by the permeation experiment on the Caco-2 monolayer, was also associated with an increase in bioavailability, a phase 1 clinical trial was set up involving 5 healthy adult volunteers using a crossover double-blind design (Q10-CROSS Study; Bioethical Committee approval prot. no. 0302426 10/06/2024, conducted in the Department of Medical Sciences, University of Turin).

The subjects were selected in accordance with the following inclusion and exclusion criteria:

- Inclusion criteria
- Age between 20 and 60 years;
- No medications taken in the last 15 days;
- Informed consent obtained.
- Exclusion criteria
- Participation in competitive sports;
- BMI > 25;
- Ongoing chronic inflammatory disease (chronic autoimmune diseases such as rheumatoid arthritis, psoriatic arthritis, ankylosing spondylitis, psoriasis, lupus, and connective tissue diseases);
- Ongoing or recently diagnosed cardiovascular disease (congestive heart failure, recent myocardial infarction, thrombophlebitis);
- Ongoing oncological disease and related anticancer treatment;
- Intake of coenzyme Q10 supplements in the last 30 days;
- Diet rich in meat (pork, beef, chicken), oily fish (herring, sardines, shellfish, mollusks) in the two weeks prior to the trial.

#### 2.14.1. Dietary Recommendations

Participants were advised to follow the following guidelines:

✓ Avoid foods rich in coenzyme Q10 for the two weeks prior to and during the trial including:
  ○ Meat (pork, beef, chicken);
  ○ Oily fish (such as herring, sardines, mackerel);
  ○ Shellfish and mollusks.
✓ Limit intake of foods high in antioxidants like the following:
  ○ Berries (blueberries, strawberries, raspberries);
  ○ Leafy green vegetables (spinach, kale);
  ○ Nuts and seeds (walnuts, almonds).
✓ Maintain a consistent diet to avoid fluctuations in bioavailability that could affect the study outcomes. Avoid introducing new foods or significant dietary changes during the study period.
✓ Avoid dietary supplements or multivitamins containing coenzyme Q10 or other antioxidants for at least 30 days prior to the trial and throughout the duration of the study.
✓ Stay hydrated by drinking adequate amounts of water daily, as dehydration could influence absorption and blood sampling results.
✓ Limit alcohol intake to avoid interference with the metabolism and absorption of CoQ10.
✓ Balanced meals: follow a balanced and regular eating schedule, with no extreme fasting or overeating, to maintain stable metabolic conditions during the study.
✓ After 2 h from intake of CoQ10 test product, volunteers were allowed a light breakfast without fatty foods (butter, oil, peanuts, milk to avoid any interference with CoQ10 bioaccessibility).

#### 2.14.2. Scheduled Visits

- T0 (Day 0): screening visit + delivery of dietary recommendations, randomization visit.
- T1 (Day 15): first administration of CoQ10 product.
- Venous blood sampling at t = 0 min, t = 60 min, t = 120 min, t = 240 min, t = 360 min, t = 720 min.
- T2 (Day 45): second administration of CoQ10 product.
- Venous blood sampling at t = 0 min, t = 60 min, t = 120 min, t = 240 min, t = 360 min, t = 720 min.

#### 2.14.3. Data Collected

- Demographic data (T0);
- Medical and pharmacological history (T0);
- Weight/height/BMI.

#### 2.14.4. Pharmacokinetic Parameters

C_max_, T_max_, AUC_0–t_, ΔAUC_0–t_, ΔC_max_.

#### 2.14.5. Bioanalytical Method

Ubiquinone quantification was performed in lithium/heparin anticoagulated plasma from healthy volunteers using a high-performance liquid chromatography (HPLC) method with ultraviolet (UV) absorbance detection.

Specifically, samples were analyzed using a CE-IVD bioanalytical kit (Chromsystems, code 68000) for measurement of ubiquinone in plasma or whole blood.

#### 2.14.6. Sample Preparation

Sample preparation followed the protocol and involved using 500 µL of plasma transferred into amber reaction tubes. After adding 250 µL of a solution containing the internal standard (IS, a molecule with chemical characteristics similar to ubiquinone but with a different HPLC retention time), a two-step protein precipitation procedure was performed using different precipitation solvents, followed by solid-phase extraction (SPE). Below are the details of the two purification steps:

- Protein precipitation
- Step 1: 500 µL of “precipitation reagent 1” was added to the sample containing IS, mixed by vortexing for 30 s.
- The samples were incubated at 4 °C in the dark for 10 min to facilitate protein precipitation, then centrifuged at 15,000× *g* for 5 min in a refrigerated centrifuge to obtain a solid pellet.
- Step 2: 100 µL of “precipitation reagent 2” was added to the same tubes, mixed by vortexing for 30 s, and centrifuged again at 15,000× *g* for 10 min in a refrigerated centrifuge.
  At that stage, a “clarified” supernatant free of plasma proteins was obtained.
- Solid-Phase Extraction (SPE)

Following protein precipitation, the supernatant of each sample was transferred to a dedicated solid-phase extraction column. All steps were performed in environments shielded from sunlight and under minimal indirect ambient lighting to minimize potential photodegradation of ubiquinone.

- Columns, placed in glass collection tubes, were centrifuged at 700× *g* for 2 min to remove plasma contaminants and precipitation solvents (which were discarded), retaining ubiquinone within the solid-phase chromatographic matrix.
- This operation was repeated twice, using 500 µL of “washing buffer 1” and 160 µL of “washing buffer 2” to remove most matrix contaminants.
- Finally, columns were transferred to new collection tubes, and 250 µL of “elution solvent” was added to release ubiquinone from the chromatographic matrix, yielding the purified extract.

The resulting extracts were immediately transferred to amber chromatographic vials and placed in the analytical system.

#### 2.14.7. Chromatographic Separation and Data Processing

The chromatographic system used was a UHPLC-PDA (photodiode array detector, multi-wavelength UV detector, Waters, 102 Tide Mill Road, Hampton, NH, USA) operated in HPLC-UV mode per protocol.

- The system was first decontaminated with isopropanol and water (both at 0.4 mL/min for 1 h).
- Then, the system was loaded with the mobile phase provided with the chromatographic kit for 30 min at 0.4 mL/min to remove traces of other solvents.
- The analytical column, equipped with a pre-column, was equilibrated with the mobile phase for 1 h at the working flow rate (2.5 mL/min) before starting the analysis.

The chromatographic method employed an isocratic reverse-phase separation, where the composition of the mobile phase remained constant throughout the analysis.

- Flow rate: 2.5 mL/min, thermostat set at 30 °C.
- Run time: 14 min.
- Injection volume: 50 µL.
- Sampling needle wash solvent: isopropanol, as indicated in the kit.
- Ubiquinone chromatograms were acquired by monitoring UV absorbance at 275 nm, with IS and ubiquinone peaks observed at 7.5 and 12.5 min, respectively.
  Analytical results were processed using Empower 2 software (Waters, 102 Tide Mill Road, Hampton, NH, USA).
- Calibration was performed by repeatedly injecting the calibration standard until a consistent peak area was achieved, using a forced linear fitting through the origin of the axes. The quantification signal (“response”) was the ratio between the ubiquinone and IS peak areas.
- At each analytical session, the two quality controls provided with the kit were injected at the beginning and end of the session to verify analytical performance within EMA guideline acceptance ranges (maximum 15% deviation from nominal value for accuracy, and maximum 15% relative standard deviation for precision). These criteria were met in all sessions. All samples were analyzed in two independent analytical sessions, alternating samples from the first and second evaluations (blinded to the treatment received by the patients) to minimize the effects of minor intra-session analytical variations.

### 2.15. Statistical Analyses and Calculations

All the data reported are expressed as mean +/− SD of three different experiments. Student’s *t*-test was used within pairs of experiments using a one- or two-tail test. The analysis of variance was performed with one-way ANOVA test. A post hoc Duncan test was also performed. For all analyses, *p* < 0.05 was considered to express statistical significance.

For the clinical trial, the FDA-EMA limit of 80 < AUC < 125 with 90% CI of the logarithmic ratio of the geometric mean was chosen as the statistical test to compare the bioavailability of the two formulations (this test is equal to two-one sided *t*-test with the null hypothesis of bioequivalence at the 5% significance level according to FDA-EMEA guideline on the investigation of bioequivalence). To compare the percentage of absorption, the following equation was used:

%absorption = (C_max_ × V_plasma_*)/dosage taken (g) × 100

* V_plasma_ is conventionally set at 2.5 L.

The following statistical analyses were also conducted:

- A complete descriptive analysis of all parameters studied: mean, geometric mean, standard deviation, coefficient of variation, 95% confidence interval, normality test (Shapiro–Wilk);
- A power of 80% for *n* = 5 was considered sufficient according to the following equation:

*n* > 2[(Z_a_/2 + *Z_b_*) × *s*/*d*]^2^

*Z_a_* = 1.96      for a = 5%

Z_b_ = 0.842, 1.282, 1.645     for power = 80, 90, 95%

where *n* is the sample size, *Z_b_* is the desired power, *Z_a_*/2 represents the level of statistical significance, *s*^2^ is the standard deviation of the outcome variable, and *d*^2^ is the difference in means. The effect size, expressed as Cohen’s d, was calculated as well. The software used were Phoenix WinNonLin 8.4 software (Certara, Hyderabad, Telangana, India) and SPSS 29.0 software (IBM, Armonk, NJ, USA) through non-parametric Wilcoxon tests for paired samples. The significance threshold was conventionally set with α (Type I error probability) at 0.05.

## 3. Results and Discussion

### 3.1. Dispersed Phase Size in FaSSIF by Dynamic Light Scattering (DLS)

**Figure 1:**
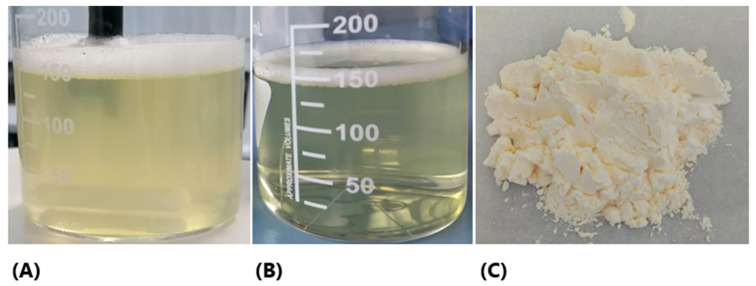
Dispersion of CoQ10 LiBADDS 027 in water (**A**) and FaSSIF (**B**). It is possible to appreciate the greater clarity of the dispersion in FaSSIF compared to that in water. (**C**) CoQ10 LiBADDS powder.

The dispersion of CoQ10 LiBADDS in water and FaSSIF after simulated digestion resulted in a translucent system in the first case and an almost clear system in the second, as shown in Figure 1.

**Figure 2:**
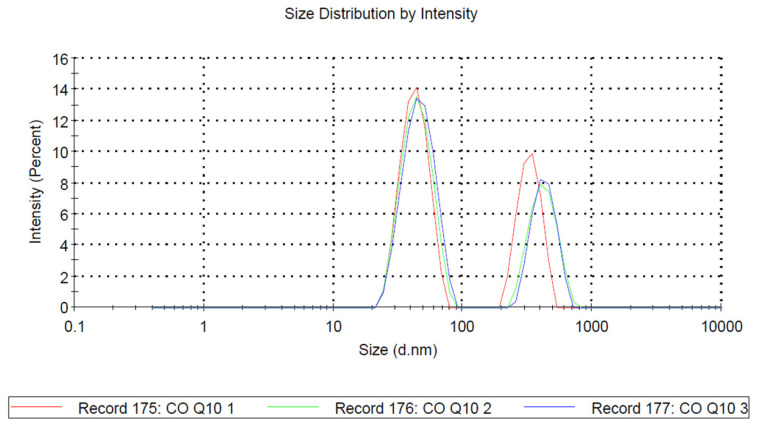
Average size of mixed micelles of CoQ10 LiBADDS 028 dispersed in FaSSIF.W.

The enhanced clarity of the system in digestive fluids was facilitated by bile salts, which reduced the average droplet size of the dispersed oil phase by acting as co-surfactants. The size analysis of the CoQ10 LiBADDS 028 dispersion in FaSSIF, conducted using DLS, revealed the presence of a nanoemulsion with a primary peak composed of particles with an average diameter of approximately 43 nm, and a secondary, less prominent peak composed of particles with a diameter around 330 nm (Figure 2). The polydispersity index (PDI) was relatively high (Figure 1; Table 2).

It is noteworthy that dispersion occurred in FaSSIF at 37 °C, suggesting that the formation of the nano-sized emulsion was spontaneous at physiological temperatures. This phenomenon was attributed to the presence of various active surfactants, such as PS80, CLA, and bile salts, which work together to increase the elasticity of the droplets, thereby reducing surface tension in accordance with Laplace’s law. CLA became an active surface agent with a negative charge above pH 6, as it was present in its carboxylic form. The negative z potential confirmed a deposition of negatively charged surfactants around oily droplets represented by CLA and SOS, both in ionic form, at the pseudo-neutral pH of the small intestine. Nanoemulsions, even according to the low-energy method, usually take place at 70 °C or higher and with high shear rate mixing [37]. In our case, the technological form projected was a SEDDS that generated a nanoemulsion at physiological temperatures although with a relatively high polydispersity index because of the low energy supply. We could have improved the distribution uniformity by introducing a larger amount of PS80 that, on the other hand, would have worsened the taste and increased toxicity on the intestinal epithelium. Taking into account these data and the average dimension of the most relevant peak around 40 nm, it would suggest the formation of a microemulsion rather than a nanoemulsion, and this hypothesis is further supported by the evidence that such dispersion forms spontaneously, in the absence of an energy output even minimally sufficient to generate nanoemulsions.

### 3.2. Bioaccessibility of CoQ10 LiBADDS Dispersion in FaSSIF

According to Equation (1), the bioaccessibility corresponding to the BF of CoQ10 from unformulated CoQ10 and CoQ10 LiBADDS 021, 022, 023, 024, 025, 026, 027, 028 in FaSSIF was 13%, 18%, 20%, 20%, 74%, 96%, 96%, 93%, and 77%, respectively (Table 3).

The production of dispersible powder does not face the technical challenges typically associated with tablet production and, as a result, allows for the introduction of an adequate amount of PS80, which is required to effectively solubilize CoQ10, along with a certain amount of MCT, where applicable. Accordingly, different powders were produced with increasing PS80/CoQ10 ratios, with or without MCT, and with or without NaC, to determine the minimal effective PS80/CoQ10 ratio capable of enhancing the bioavailability (BF) of CoQ10. The presence of MCT was found to drive the BF of CoQ10. As shown in Table 3, the highest CoQ10 BF, ranging from 74 to 96%, was achieved with CoQ10 LiBADDS 024, 025, 026, 027, and 028, all of which contained MCT. Another key observation from the analysis of the formulations based on CoQ10’s BF is that NaC did not play a significant role in modifying the BF. In fact, CoQ10 LiBADDS 021, 022, and 023, which contained NaC, showed a very low BF compared to those without NaC. From the formulation analysis, it can be concluded that MCT plays a crucial role in improving CoQ10’s BF, as all formulations lacking MCT resulted in a low BF, even with a higher PS80/CoQ10 ratio. Specifically, for CoQ10 LiBADDS 023, increasing the PS80/CoQ10 ratio to 10, in the absence of MCT, did not lead to a significant improvement in CoQ10 BF compared to CoQ10 LiBADDS 021 and 022, with PS80/CoQ10 ratios of six and eight, respectively. Interestingly, CoQ10 LiBADDS 028, which did not contain PS80 but had the same amount of MCT, CLA, and SOS as CoQ10 LiBADDS 027, achieved a BF greater than 75%, suggesting that the combination of MCT, CLA, and SOS alone is sufficient to ensure a high BF of CoQ10. Taken together, these data support the hypothesis that CoQ10 in a solubilized phase containing PS80 in a 1:1 ratio with CoQ10, along with CLA and MCT, adsorbed in a mixture of maltodextrins and SOS (CoQ10 LiBADDS 027), provides the optimal conditions for achieving a rapid emulsification with high bioaccessibility of CoQ10.

### 3.3. Integrity of Barrier Function in the Model of Enteric Epithelium

**Figure 3:**
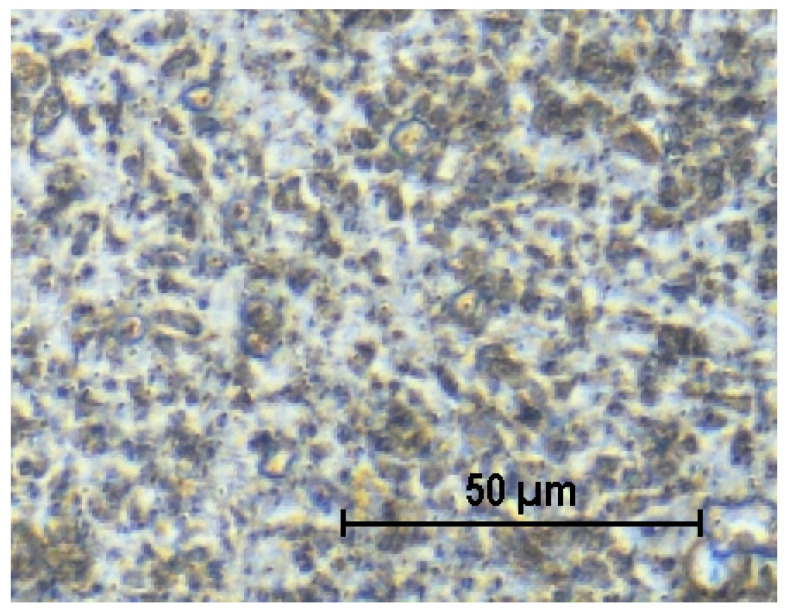
Caco-2 cell insert.

The TEER values of the monolayer recorded in all wells before applying the formulations were over 350 ohm/cm^2^, which reflected the firmness and integrity of the monolayer. The further determination of the LY passage through the monolayer after 1 h gave a concentration very close to zero, and *P_app_*_(cm/s)_ was lower than 1 × 10^−8^, which confirmed a remarkable integrity of Caco-2 tight junctions (Figure 3).

The TEER values of the monolayer after 1, 2, and 3 h since application in the apical side of the CoQ10 LiBADDS 027 formulation at its maximal nontoxic concentration suggested maintained monolayer integrity (Table S1 and Figure S2 are available in Supplementary Materials), confirming that the formulation did not produce any significant perturbance of the monolayer permeability. The same profile could be described for the application of CoQ10 027 on the BL side. All TEER measurements were far higher than the recognized lower threshold of 200–250 Ohm/cm^2^. Similar considerations could be drawn regarding unformulated CoQ10. The difference in terms of reduction in TEER between the CoQ10 LiBADDS 027 formulation and unformulated CoQ10 at times 1, 2, 3, and 24 h was inconsistent, suggesting that the CoQ10 LiBADDS formulation did not significantly affect the monolayer integrity. The *P_app_* (cm/s) of LY calculated after the application of CoQ10 LiBADDS 027 and unformulated CoQ10 at 3 h were very close to those at baseline, thereby confirming that the LiBADDS formulation did not affect the tight junctions’ integrity of the monolayer. The dispersed formulation of CoQ10 (CoQ10 LiBADDS 027) in digestive fluids was subjected to increasing dilution with HBSS to assess the maximal nontoxic concentration to be applied to the enteric monolayer. The dilution 1:1 was not toxic for the monolayer, which allowed us to maintain a TEER value higher than 350 Ohm/cm^2^ after 1 h from the sample application.

### 3.4. CoQ10 Permeation

The permeable fraction of CoQ10 from both CoQ10 (unformulated) and CoQ10 (LiBADDS 027) formulations can be calculated based on the CoQ10 fraction remaining in the supernatant bulk (BF) after simulated digestion (FSSGF + FaSSIF) and centrifugation. The BF represents the water-dispersible CoQ10 that is capable of fully permeating through the enteric epithelium, thus leading to its absorption. In contrast, the so-called “pellet” non-dispersible fraction of CoQ10, which results from the centrifugation of the digested dispersion, represents the insoluble CoQ10 fraction that cannot permeate through the intestine. For this reason, it was not tested for absorption using the Caco-2 cell intestinal monolayer. Permeability was therefore calculated by multiplying the BF by the internalization rate (IR), according to Equation (4):

%permeability CoQ10_(LiBADDS 027)_ = BF(CoQ10_(LiBADDS 027)_ × % IR CoQ10)/100 (4)

38.4% +/− 6.32% of the initial dose internalized by Caco-2 cells

%permeability CoQ10_(Unformulated)_ = BF(CoQ10_(Unformulated)_ × % IR CoQ10)/100

3.68% +/− 1.10% of the initial dose internalized by Caco-2 cells

The results reported in Table 4 highlight a statistically significant difference of 38% (*p* < 0.01) between the LiBADDS formulation and unformulated CoQ10 in terms of CoQ10 internalization, confirming a higher rate of internalization for the former. This is a crucial aspect for improving CoQ10 bioavailability, as the efficiency of intracellular transport is often a limitation for lipophilic compounds like CoQ10. The data relating to the BF confirmed the poor water dispersion of CoQ10 in FSSGF/FaSSIF despite the presence of bile salts, while CoQ10 delivery in LiBADDS significantly improved the bioaccessibility of CoQ10 by increasing its dispersibility in water. Regarding the Caco-2 monolayer permeability and cellular internalization, the findings suggested a high rate of internalization for both the BF of CoQ10 LiBADDS and that of the unformulated form, although the latter occurred at a rate significantly lower than LiBADDS (Table 4) in accordance with the linear correlation between the water dispersibility of CoQ10 and Caco-2 intracellular uptake rate described by Bhagavan [24]. It was not possible to calculate *P_app_* and the permeation rate for both CoQ10 LiBADDS and unformulated CoQ10 and for both permeation directions, because the concentration in the acceptor compartment was not detectable in HPLC. A possible explanation for this could be that CoQ10 interacted with the albumin present in the HBSS [38,39]. However, the experiment conducted with HBSS deprived of BSA and supplemented with magnesium and calcium salts yielded the same result. The only possible explanation, therefore, was that the amount of CoQ10 reaching the acceptor compartment was lower than the LOQ. These data are consistent with the published papers and with the physiology of CoQ10 absorption, which is massively transported into the lymph and therefore is minimally delivered into the blood circulation after intestinal permeation.

### 3.5. Bioavailability Assessment

**Figure 4:**
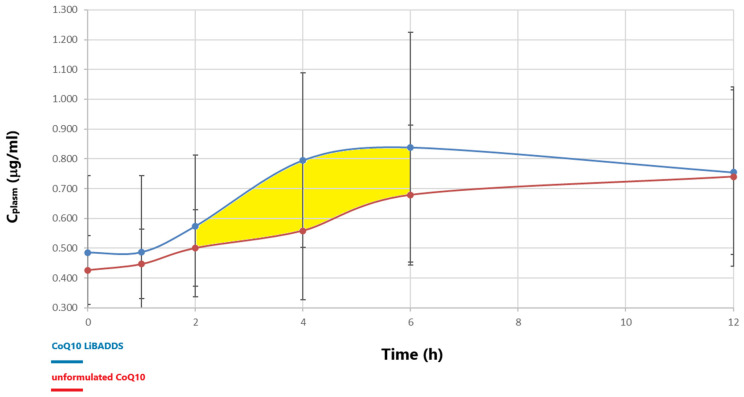
Overlapping graphs of unformulated CoQ10 and LiBADDS Co10 plasmatic concentrations versus time (h) after oral administration of 60 mg CoQ10 in fasting conditions (*n* = 5). In yellow, the ΔAUC_2–6h_ is evidenced.

The differences in both the means of the plasma concentrations and AUC at 1, 2, 4, 6, and 12 h between CoQ10 LiBADDS and unformulated CoQ10 were statistically significant (*t*-test, α < 0.05; Figure 4).

From the analysis and comparison of the main pharmacokinetic parameters, particularly C_max_ and the AUC, the LiBADDS formulation demonstrated higher bioavailability compared to the unformulated CoQ10 formulation. The logarithm of the geometric mean ratio of AUC_0–12h_ and AUC_2–6h_ between the LiBADDS formulation and unformulated CoQ10 was 126 and 141, respectively, indicating superior bioavailability according to the criteria set by FDA-EMA (Table 5).

These guidelines establish that bioequivalence between two different formulations should be assessed through the ratio of the logarithms of the geometric means of their respective AUCs, with this ratio falling within the 80–125% range, as indicated by a 90% confidence interval [40]. The FDA-EMA document also specifies that this statistical tool is equivalent to a two one-sided *t*-test (TOST), with the null hypothesis of bioequivalence tested at a 5% significance level. The same test was applied to compare C_max_. The logarithmic geometric mean ratio of Cmax between the LiBADDS formulation and unformulated CoQ10 was 126. T_max_ was 17% shorter for the LiBADDS formulation compared to the unformulated CoQ10 (Table 5). These results are consistent with previously published data regarding CoQ10 solubilized in SEDDS formulations and cyclodextrins [41]. A high variability in plasma concentrations at t = 0 was observed, consistent with published studies, due to the constant presence of CoQ10 in plasma and significant variability in dietary intake, despite dietary restrictions imposed on participants two weeks prior to sampling. Plasma concentration at t = 0 was set at the lowest value observed within the first two hours, eliminating values below C_0_, which could have been influenced by physiological fluctuations in CoQ10 concentrations before reaching systemic circulation. The AUC was calculated using the trapezoidal method for both the 0–12 h and 2–6 h intervals, with the latter showing the greatest uniformity in the AUC curve trend. Extrapolation of the AUC to infinity was not possible, as T_last_ was highly variable between subjects, with negative values in three out of five patients. Plasma concentrations at t = 12 h were highly heterogeneous, with a rising trend in some patients, suggesting a two-peak trend, likely due to entero-hepatic recirculation, as indicated in published studies [42]. The absorption percentage was 4.3% ± 0.6% for the LiBADDS formulation and 3.5% ± 0.5% for the unformulated CoQ10 formulation, indicating a 23% increase in absorption for the LiBADDS formulation compared to the unformulated CoQ10. Finally, ΔAUC_0–12h_ and ΔAUC_2–6h_ were 131 and 206, respectively (Table 5), confirming a significant difference in bioavailability between CoQ10 LiBADDS and unformulated CoQ10, especially in the 2–6 h interval in favor of the LiBADDS formulation. These results are consistent with those published by Pravst [27]. The ΔC_max_ ratio between the two formulations was 116 (Table 5). Effect size, expressed as Cohen’s d, was greater than 1 for ΔAUC_0–12_, ΔAUC_2–6_, and AUC_2–6_; greater than 0.5 but less than 0.8 for AUC_0–12_, AUC_2–6_, and C_max_; and greater than 0.2 but less than 0.5 for ΔC_max_ and T_max_. The missing delayed peak curve, typical of nanoemulsion, is of particular interest. A possible explanation lies in the lymphatic access of CoQ10 in the enterocyte: this peculiar mechanism could interfere with the common mechanism of delivery of CoQ10 fostered by nanoemulsions encapsulating molecules that are delivered into the portal circulation. Lastly, it is very likely that the dispersion generated in the intestine herein described resembles more closely a microemulsion rather than a nanoemulsion since the main peak was around 40 nm and the dispersion occurred at physiological temperatures and without any strong mixing supply. Microemulsions, unlike nanoemulsions, are spontaneously generating dispersions with average dimensions lower than 100 nm. In this case, a spontaneous, bicontinous, mesotrophic phase occurs and does not need any energy output according to Gibbs’s Law.

## 4. Conclusions

A LiBADDS system was developed in which the combination of MCT, PS80, and an SOS derivative significantly enhanced the bioaccessibility of CoQ10 compared to its unformulated counterpart, following simulated digestion in FSSGF and FaSSIF. Notably, MCT played a pivotal role in enhancing bioaccessibility, as the presence of only the non-ionic surfactant and SOS was insufficient to achieve the same level of improvement. This system also facilitated the internalization of CoQ10 into intestinal cells, as demonstrated in the Caco-2 model, compared to unformulated CoQ10. The CoQ10 LiBADDS formulation displayed exceptional dispersibility in water upon simple mixing, with this effect being even more pronounced in the FaSSIF environment. This phenomenon is likely attributable to the formation of a spontaneous microemulsion upon contact with intestinal fluids, as confirmed by the extremely small size of the oily droplets of the main peak detected by DLS. Furthermore, a crossover, double-blind clinical trial involving five healthy volunteers showed that the LiBADDS formulation significantly increased CoQ10 intestinal absorption, C_max_, and AUC_2–6h_, suggesting that this technology could play a critical role in improving CoQ10 bioavailability. However, to confirm these findings and provide more robust data, a larger clinical trial involving a higher number of healthy participants should be conducted. This trial should use a crossover design and assess CoQ10 bioavailability over extended periods (0–24 h and 0–48 h), allowing for a more comprehensive evaluation of the real impact of the LiBADDS technology on CoQ10 bioavailability. Such a study would offer deeper insights into the long-term benefits and potential applications of the LiBADDS in enhancing the bioavailability of CoQ10, making it a promising candidate for future therapeutic and supplementation formulations.

## 5. Patents

The technology and formulations herein described have been patented (N° 102024000029277) by University of Padua and by Laboratorio della Farmacia SPA, Quarto d’Altino, Venice, Italy with 50% joint ownership.

## Figures and Tables

**Table 1:** Quali-quantitative composition per single CoQ10-LiBADDS sachet.

	CoQ10 LiBADDS 028 1 Sachet	CoQ10 LiBADDS 027 1 Sachet
CoQ10 (g)	0.0200	0.020
Labrafac lipophile WL 1349 (MCT) (g)	0.0400	0.040
Cleargum CO 001 (SOS)	0.0500	0.0500
Veremul T 80 (PS 80) g	0.1200	0.1200
Glucidex 19 D (g)	Up to 5.0 g	Up to 5.0 g
Emul AC (g)	0.5000	0
Tonalin A 80 (CLA) (g)	0.0020	0.0020
Tocopherol acetate 66% (g)	0.0025	0.0025
Amorphous silica (g)	0.3500	0.3500
Total (g)	5.0000	5.0000

**Table 2:** Average size of nano-micelle generated from CoQ10 LiBADDS 028 in FaSSIF, ζ potential, and relative PDI.

		Size (d.nm)	% Intensity	St Dev (d.nm)	ζ Potential
Z-Average (d.nm): 128.4	Peak 1:	43.24	62.7	10.15	−18.24 Mv+/−3.67
PDI: 0.373	Peak 2:	329.8	37.3	65.35	
Intercept: 0.964	Peak 3:	0.000	0.0	0.000	

**Table 3:** BF (bioaccessible fraction) of CoQ10 in the mixed micelle/emulsion generated from the dispersion of unformulated CoQ10, CoQ10 LiBADDS 021, 022, 023, 024, 025, 026, 027, 028 in FaSSIF.

	CoQ10 Unform.	CoQ10 LiBADDS 021 (Sachet)	CoQ10 LiBADDS 022 (Sachet)	CoQ10 LiBADDS 023 (Sachet)	CoQ10 LiBADDS 024 (Sachet)	CoQ10 LiBADDS 025 (Sachet)	CoQ10 LiBADDS 026 (Sachet)	CoQ10 LiBADDS 027 (Sachet)	CoQ10 LiBADDS 028 (Sachet)
CoQ10 (theoretical) (g)	0.020	0.020	0.020	0.020	0.020	0.020	0.020	0.020	0.020
CoQ10 (measured) (g)	0.019 +/− 0.0022	0.019 +/− 0.0026	0.019 +/− 0.0026	0.019 +/− 0.0036	0.019 +/− 0.0034	0.019 +/− 0.0020	0.019 +/− 0.0029	0.019 +/− 0.0034	0.019 +/− 0.0030
oxCoQ10	0.0078 +/− 0.0005	0.0076 +/− 0.0006	0.0077 +/− 0.0006	0.0078 +/− 0.0004	0.0078 +/− 0.0005	0.0079 +/− 0.0004	0.0078 +/− 0.0004	0.0080 +/− 0.0006	0.0074 +/− 0.0003
rCoQ10	0.011 +/− 0.0003	0.011 +/− 0.0004	0.012 +/− 0.0005	0.010 +/− 0.0004	0.012 +/− 0.0003	0.011 +/− 0.0004	0.012 +/− 0.0002	0.012 +/− 0.0003	0.011 +/− 0.0006
BF (%)	13.0 +/− 1.10	17.6 +/− 1.42	19.8 +/− 2.41	20.4 +/− 3.41	73.8 +/− 8.68	96.5 +/− 8.48	96.4 +/− 10.31	92.8 +/− 9.11	76.8 +/− 8.88
Ratio PS80/CoQ10	-	6	8	10	8	4	2	1	0
NaC	-	0.100	0.100	0.100	-	-	-	-	-
Labrafac Lipophile WL 1379 (MCT) g	-	-	-	-	0.020	0.040	0.040	0.040	0.040
Cleargum CR 01 (SOS)		0.100	0.100	0.100	0.100	0.100	0.100	0.100	0.100
Tonalin A 80 (CLA) g	-	0.020	0.020	0.020	0.020	0.020	0.020	0.020	0.020
Industrial machinability	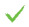	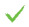	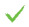	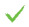	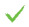	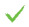	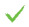	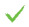	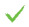

**Table 4:** *P_app_*, % permeation, internalization rate, efflux rate, and mass balance of CoQ10 from CoQ10 (LiBADDS 027) in the apical to basolateral side direction and basolateral to apical direction after 3 h. The results are expressed as the mean of three different experiments +/− SD.

CoQ10 LiBADDS 027 (Ap 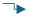 Bl) C = 0.030 mM	CoQ10 LiBADDS 027 (Bl 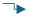 Ap) C = 0.030 mM
*P_app_* Not detectable C CoQ10 intracellular 24.3 +/− 2.6 nmoles/mg proteins %internalization (IR) (A-B) (3 h) 39.2 +/− 5.3 BF (%) 93.8 +/− 10.2 Mass balance 83.7 +/− 10.5	*P_app_* Not detectable C CoQ10 intracellular 36.6 +/− 3.1 nmoles/mg proteins %internalization (IR) (B-A) (3 h) 51.2 +/− 8.6 Mass balance 78.3 +/− 12.9
Unformulated CoQ10 (Ap 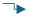 Bl) C = 0.030 mM	Unformulated CoQ10 (Bl 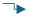 Ap) C = 0.030 mM
P_app_ Not detectable C CoQ10 intracellular 14.4 +/− 2.8 nmoles/mg proteins %internalization (IR) (A-B) (3 h) 28.3 +/− 4.4 BF (%) 13.0 +/− 1.10 Mass balance 80.4 +/− 14.2	P_app_ Not detectable C CoQ10 intracellular 20.9 +/− 3.9 nmoles/mg proteins %internalization (IR) (B-A) (3 h) 44.6 +/− 3.6 Mass balance 82.6 +/− 11.1

**Table 5:** Summary of pharmacokinetic parameters (*n* = 5).

Parameter (Mean) +/− SD	C_max (mg)_ CI 95%	DC_max (mg)_ CI 95%	AUC_0−12h (mg/L/h)_ Cl 95%	DAUC_0−12h (mg/L/h)_ CI 95%	AUC_2−6h (mg/L/h)_ Cl 95%	DAUC_2−6h (mg/L/h)_ Cl95%	T_max (h)_ Cl95%
CoQ10 LiBADDS Unform. CoQ10	1.08 +/− 0.15	0.33 +/− 0.18	10.2 +/− 4.2	4.38 +/− 0.16	4.12 +/− 1.55	1.15 +/− 0.17	6.8 +/− 3.03
	(0.93–1.23)	(0.15–0.51)	(6.08–14.3)	(4.22–4.54)	(2.60–5.64)	(0.98–1.32)	(3.83–9.77)
	0.85 +/− 0.09	0.29 +/− 0.06	7.9 +/− 2.1	3.34 +/− 0.55	2.86 +/− 0.62	0.56 +/− 0.35	7.6 +/− 4.34
	(0.76–0.94)	(0.23–0.35)	(5.84–9.96)	(2.80–3.88)	(2.25–3.45)	(0.22–0.90)	(3.35–11.85)
Ratio for mean % (CoQ10 LiBADDS)/(Unform.CoQ10)		114		131		205	
Ratio for mean % Log geometric mean CI 90% 80–125% (FDA/EMEA guidelines) (CoQ10 LiBADDS)/(Unform. CoQ10)	126		126		141		

## Data Availability

Research data are not available because a patent has been filed.

## Supplementary Materials

The following supporting information can be downloaded at: https://www.mdpi.com/article/10.3390/pharmaceutics17040414/s1, Figure S1: The standard calibration curve was constructed by diluting CoQ10 in MeOH, to give final concentrations of calibrator solutions in the range of 0.5 to 150 mg/mL.; Figure S2: Histograms representing TEER values before and after application of CoQ10 LiBADDS 027 and CoQ10 in both the apical and basolateral side of Transwell plates containing Caco-2 monolayer at 1, 2, 3, and 24 h. The values are expressed as the mean +/– SD of three different experiments.; Table S1: Values of TEER recorded in the monolayer at time 0 and after 1, 2, and 3 h since the application of both the CoQ10 LiBADDS 027 and CoQ10 in the apical and basolateral side of Transwell plates. The values are expressed as the mean +/− SD of three different experiments.

## References

[1] Martelli A., Testai L., Colletti A., Cicero A.F.G. (2020). Coenzyme Q_10_: Clinical Applications in Cardiovascular Diseases. Antioxidants.

[2] Fischer A., Onur S., Niklowitz P., Menke T., Laudes M., Rimbach G., Döring F. (2016). Coenzyme Q10 Status as a Determinant of Muscular Strength in Two Independent Cohorts. PLoS ONE.

[3] Yen C.H., Chang P.S., Chang Y.H., Lin P.T. (2022). Identification of Coenzyme Q10 and Skeletal Muscle Protein Biomarkers as Potential Factors to Assist in the Diagnosis of Sarcopenia. Antioxidants.

[4] Chong E.G., Lee E.H., Sail R., Denham L., Nagaraj G., Hsueh C.T. (2021). Anthracycline-induced cardiotoxicity: A case report and review of literature. World J. Cardiol..

[5] Swain S.M., Whaley F.S., Ewer M.S. (2003). Congestive heart failure in patients treated with doxorubicin. Cancer.

[6] Kamphuis J.A.M., Linschoten M., Cramer M.J., Doevendans P.A., Asselbergs F.W., Teske A.J. (2020). Early- and late anthracycline-induced cardiac dysfunction: Echocardiographic characterization and response to heart failure therapy. Cardiooncology.

[7] Osoro I., Sharma A., Amir M., Vohra M., Kumar R., Kumar H., Zargar A., Bangar H. (2022). Prevention and management of anthracycline induced cardiotoxicity: A review. Health Sci. Rev..

[8] Ferrera A., Fiorentini V., Reale S., Solfanelli G., Tini G., Barbato E., Volpe M., Battistoni A. (2023). Anthracyclines-induced cardiac dysfunction: What every clinician should know. Rev. Cardiovasc. Med..

[9] Childs A.C., Phaneuf S.L., Dirks A.J., Phillips T., Leeuwenburgh C. (2002). Doxorubicin treatment in vivo causes cytochrome C release and cardiomyocyte apoptosis as well as increased mitochondrial efficiency superoxide dismutase activity and Bcl-2 Bax ratio. Cancer Res..

[10] Wang G.W., Klein J.B., Kang Y.J. (2001). Metallothionein inhibits doxorubicin-induced mitochondrial cytochrome c release and caspase-3 activation in cardiomyocytes. J. Pharmacol. Exp. Ther..

[11] Wallace K.B., Sardão V.A., Oliveira P.J. (2020). Mitochondrial determinants of doxorubicin-induced cardiomyopathy. Circ. Res..

[12] Vincent D.T., Ibrahim Y.F., Espey M.G., Suzuki Y.J. (2013). The role of antioxidants in the era of cardio-oncology. Cancer Chemother. Pharmacol..

[13] Qu H., Guo M., Chai H., Wang W.T., Gao Z.Y., Shi D.Z. (2018). Effects of coenzyme Q10 on statin-induced myopathy: An updated meta-analysis of randomized controlled trials. J. Am. Heart Assoc..

[14] Kennedy C., Köller Y., Surkova E. (2020). Effect of Coenzyme Q10 on statin-associated myalgia and adherence to statin therapy: A systematic review and meta-analysis. Atherosclerosis.

[15] Banach M., Serban C., Sahebkar A., Ursoniu S., Rysz J., Muntner P., Toth P.P., Jones S.R., Rizzo M., Glasser S.P. (2015). Lipid and Blood Pressure Meta-analysis Collaboration Group. Effects of coenzyme Q10 on statin-induced myopathy: A meta-analysis of randomized controlled trials. Mayo Clin. Proc..

[16] Wei H., Xin X., Zhang J., Xie Q., Naveed M., Kaiyan C., Xiao P. (2022). Effects of coenzyme Q10 supplementation on statin-induced myopathy: A meta-analysis of randomized controlled trials. Ir. J. Med. Sci..

[17] Banach M., Serban C., Ursoniu S., Rysz J., Muntner P., Toth P.P., Jones S.R., Rizzo M., Glasser S.P., Watts G.F. (2015). Lipid and Blood Pressure Meta-analysis Collaboration (LBPMC) Group. Statin therapy and plasma coenzyme Q10 concentrations—A systematic review and meta-analysis of placebo-controlled trials. Pharmacol. Res..

[18] Qu H., Meng Y.Y., Chai H., Liang F., Zhang J.Y., Gao Z.Y., Shi D.Z. (2018). The effect of statin treatment on circulating coenzyme Q10 concentrations: An updated meta-analysis of randomized controlled trials. Eur. J. Med. Res..

[19] Bhagavan H.N., Chopra R.K. (2006). Coenzyme Q10: Absorption, tissue uptake, metabolism and pharmacokinetics. Free Radic. Res..

[20] Zaki N.M. (2016). Strategies for oral delivery and mitochondrial targeting of CoQ10. Drug Deliv..

[21] Bhagavan H.N., Chopra R.K. (2007). Plasma coenzyme Q10 response to oral ingestion of coenzyme Q10 formulations. Mitochondrion.

[22] Sahoo S., Aurich M.K., Jonsson J.J., Thiele I. (2014). Membrane transporters in a human genome-scale metabolic knowledgebase and their implications for disease. Front. Physiol..

[23] Takekawa Y., Sato Y., Yamaki Y., Imai M., Noto K., Sumi M., Takekuma Y., Iseki K., Sugawara M. (2016). An approach to improve intestinal absorption of poorly absorbed water-insoluble components via Niemann–Pick C1-Like 1. Biol. Pharm. Bull..

[24] Bhagavan H.N., Chopra R.K., Craft N.E., Chitchumroonchokchai C., Failla M.L. (2007). Assessment of coenzyme Q10 absorption using an in vitro digestion-Caco-2 cell model. Int. J. Pharm..

[25] Liu Z.X., Artmann C. (2009). Relative bioavailability comparison of different coenzyme Q10 formulations with a novel delivery system. Altern. Ther. Health Med..

[26] López-Lluch G., Del Pozo-Cruz J., Sánchez-Cuesta A., Cortés-Rodríguez A.B., Navas P. (2019). Bioavailability of coenzyme Q10 supplements depends on carrier lipids and solubilization. Nutrition.

[27] Pravst I., Rodríguez Aguilera J.C., Cortes Rodriguez A.B., Jazbar J., Locatelli I., Hristov H., Žmitek K. (2020). Comparative bioavailability of different coenzyme Q10 formulations in healthy elderly individuals. Nutrients.

[28] Beg S., Javed S., Kohli K. (2010). Bioavailability enhancement of coenzyme Q10: An extensive review of patents. Recent Pat. Drug Deliv. Formul..

[29] Petrangolini G., Ronchi M., Frattini E., De Combarieu E., Allegrini P., Riva A. (2019). A new food-grade coenzyme Q10 formulation improves bioavailability: Single and repeated pharmacokinetic studies in healthy volunteers. Curr. Drug Deliv..

[30] Niu Z., Acevedo-Fani A., McDowell A., Barnett A., Loveday S.M., Singh H. (2020). Nanoemulsion structure and food matrix determine the gastrointestinal fate and in vivo bioavailability of coenzyme Q10. J. Control. Release.

[31] Balakrishnan P., Lee B.J., Oh D.H., Kim J.O., Lee Y.I., Kim D.D., Jee J.P., Lee Y.B., Woo J.S., Yong C.S. (2009). Enhanced oral bioavailability of coenzyme Q10 by self-emulsifying drug delivery systems. Int. J. Pharm..

[32] Dash S., Xiao C., Morgantini C., Lewis G.F. (2015). New insights into the regulation of chylomicron production. Annu. Rev. Nutr..

[33] Geboers S., Stappaerts J., Tack J., Annaert P., Augustijns P. (2016). In vitro and in vivo investigation of the gastrointestinal behavior of simvastatin. Int. J. Pharm..

[34] Rakusa Z.T., Kristl A., Roskar R. (2020). Quantification of reduced and oxidized coenzyme Q10 in supplements and medicines by HPLC-UVAnal. Methods.

[35] Hubatsch I., Ragnarsson E.G., Artursson P. (2007). Determination of drug permeability and prediction of drug absorption in Caco-2 monolayers. Nat. Protoc..

[36] Tavelin S., Gråsjö J., Taipalensuu J., Ocklind G., Artursson P. (2002). Applications of epithelial cell culture in studies of drug transport. Methods Mol. Biol..

[37] Safaya M., Rotliwala Y.C. (2020). Nanoemulsions: A review on low energy formulation methods, characterization, applications and optimization technique. Mater. Today Proc..

[38] Peng X., Sun Y., Qi W., Su R., He Z. (2014). Study of the interaction between coenzyme Q10 and human serum albumin: Spectroscopic approach. J. Solut. Chem..

[39] Matsushita N., Oshima T., Takahashi H., Baba Y. (2013). Enhanced water dispersibility of coenzyme Q10 by complexation with albumin hydrolysate. J. Agric. Food Chem..

[40] (2010). Guideline on the Investigation of Bioequivalence. https://www.ema.europa.eu/en/documents/scientific-guideline/guideline-investigation-bioequivalence-rev1_en.pdf.

[41] Zmitek J., Smidovnik A., Fir M., Prosek M., Zmitek K., Walczak J., Pravst I. (2008). Relative bioavailability of two forms of a novel water-soluble coenzyme Q10. Ann. Nutr. Metab..

[42] Maciejewska-Stupska K., Czarnecka K., Szymański P. (2024). Bioavailability enhancement of coenzyme Q_10_: An update of novel approaches. Arch. Pharm..

