# Supplementary material for: Novel Lipid-Based Formulation to Enhance Coenzyme Q10 Bioavailability: Preclinical Assessment and Phase 1 Pharmacokinetic Trial
Source: Pharmaceutics. 2025 Mar 25;17(4):414. doi: 10.3390/pharmaceutics17040414 (PMC12030634; doi:10.3390/pharmaceutics17040414)
Supplement: Supplementary file 1 [file pharmaceutics-17-00414-s001.zip › pharmaceutics-3453836-supplementary.pdf]

pharmaceutics

MDPI

Supplementary materials

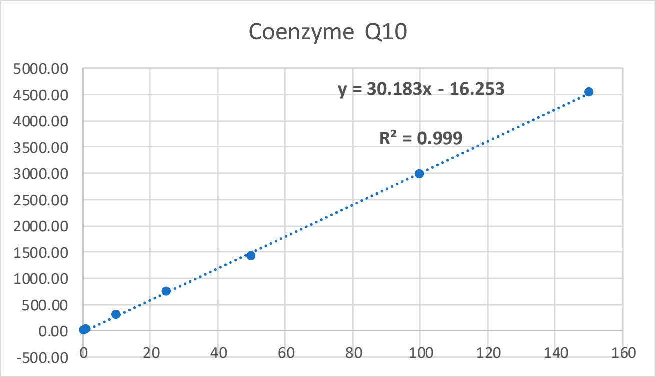

Figure S1. The standard calibration curve was constructed by diluting CoQ10 in MeOH, to give final concentrations of calibrator solutions in the range of 0.5 to 150 µg/mL.

	T0	T0 mean	SD	T 1h	T 1h mean	SD	T 2h	T 2h mean	SD	T 3h	T 3h mean	SD	T 24h	T 24 h mean	SD
Control	362.4			383.4			383.1			383.4			402.6		
	384.0	375.5	11.5	406.8	395.8	11.8	397.8	385.2	11.7	436.8	409.1	26.8	426.6	397.2	32.4
	380.1			397.2			374.7			407.1			362.4		
CoQ10	360.3			569.7			426.0			436.8			414.0		
LiBADDS	360.6	363.2	4.77	510.9	523.6	41.2	455.4	440.7	14.7	455.1	458.1	22.9	340.8	368.5	39.7
A-B	368.7			490.2			440.7			482.4			350.7		
CoQ10	452.4			424.8			582.0			435.0			313.2		
LiBADDS	393.6	411.6	35.4	371.7	403.3	27.9	519.0	538.6	37.6	408.0	429.9	19.8	338.4	337.2	23.4
B-A	388.8			413.4			514.8			446.7			360.0		
CoQ10	368.4			424.8			417.3			448.8			330.3		
A-B	399.6	397.2	27.7	371.7	385.7	34.3	535.5	440.2	86.2	517.8	473.4	38.5	394.5	374.4	35.6
	423.6			360.6			367.8			453.6			395.4		
CoQ10	374.7			421.2			413.4			426.9			296.4		
B-A	360.0	371.7	10.5	414.6	408.8	16.1	392.1	409.7	16.1	395.7	404.3	19.8	387.9	345.9	46.2
	380.4			390.6			423.6			390.3			353.4		

Table S1. Values of TEER recorded in the monolayer at time 0 and after 1, 2, and 3 h since the application of both the CoQ10 LiBADDS 027 and CoQ10 in the apical and basolateral side of Transwell plates. The values are expressed as the mean +/− SD of three different experiments.

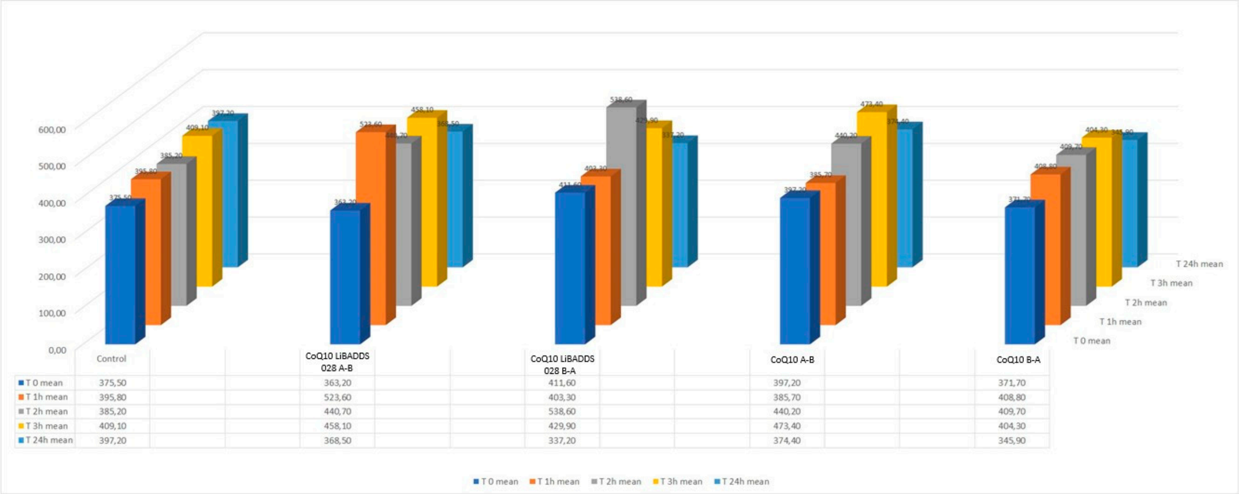

Figure S2. Histograms representing TEER values before and after application of CoQ10 LiBADDS 027 and CoQ10 in both the apical and basolateral side of Transwell plates containing Caco-2 monolayer at 1, 2, 3, and 24 h. The values are expressed as the mean +/– SD of three different experiments.
